# Case Report: A small cell lung cancer transformed from an *EGFR*-mutated Adenocarcinoma demonstrated a long-term remission to anti PD-1 antibody

**DOI:** 10.3389/fonc.2025.1651248

**Published:** 2025-09-10

**Authors:** Yusuke Kawanaka, Kimio Yonesaka, Junko Tanizaki, Osamu Maenishi, Kazuko Sakai, Kazuhiro Kakimi, Kazuto Nishio, Hidetoshi Hayashi

**Affiliations:** ^1^ Department of Medical Oncology, Kindai University Faculty of Medicine, Osaka-sayama, Osaka, Japan; ^2^ Department of Pathology, Kindai University Faculty of Medicine, Osaka-sayama, Osaka, Japan; ^3^ Department of Genome Biology, Kindai University Faculty of Medicine, Osaka-sayama, Osaka, Japan; ^4^ Department of Immunology, Kindai University Faculty of Medicine, Osaka-sayama, Osaka, Japan

**Keywords:** EGFR, EGFR-TKI, non-small cell lung cancer, small cell lung cancer transformation, case report

## Abstract

Transformation to small cell lung cancer (SCLC) is a resistance mechanism in epidermal growth factor receptor (*EGFR*)-mutated non-small cell lung cancer (NSCLC) after EGFR-tyrosine kinase inhibitor (EGFR-TKI) treatment. The efficacy of immune checkpoint inhibitor (ICI) in transformed SCLC remains to be elucidated. The present case report highlights a patient whose tumor underwent transformation to SCLC after developing resistance to an EGFR-TKI treatment. The patient subsequently achieved long-term remission lasting more than 5 years through treatment with an anti-PD-1 antibody nivolumab. Generally, the efficacy of ICI is inferior in *EGFR*-mutated NSCLC compared to those with *EGFR* wild-type NSCLC. However, some cases that have transformed to SCLC may be sensitive to ICI treatment. Further investigation is necessary to determine the efficacy of ICI in cases that have undergone transformation to SCLC.

## 1 Introduction

Epidermal growth factor receptor tyrosine kinase inhibitors (EGFR-TKIs) are the standard of care for patients with *EGFR*-mutated non-small cell lung cancer (NSCLC), and most patients achieve tumor shrinkage (1, 2). However, resistance to EGFR-TKIs eventually develops in all tumors. The mechanisms of resistance vary, including secondary *EGFR* mutations, amplification of *MET*, and transformation to small cell lung cancer (SCLC) (1). Approximately 3–10% of tumors that acquired resistance to EGFR-TKI transform to SCLC (3). The treatment of such cases is generally a combination of platinum and etoposide, which is the standard of care for patients with extensive stage SCLC (3).

Immune checkpoint inhibitors (ICIs), including PD-1/PD-L1 inhibitors, represent an established standard of care for NSCLC (4). However, the efficacy of ICI therapy is limited in patients with *EGFR*-mutated NSCLC, specifically, the response rate in a phase II trial of nivolumab monotherapy in patients after resistance to EGFR-TKI therapy was 9.6% (5). And the median progression-free survival with nivolumab was also 1.7 months (95% CI 1.3−2.3 months), which was worse than standard platinum combination chemotherapy (stratified log-rank test P = 0.001; stratified Cox 313 PH model HR of 1.92, with a 95% CI of 1.27-2.90) (5). The role of ICIs as a treatment following the development of resistance to EGFR-TKI therapy remains to be delineated (6). Conversely, in extensive SCLC, the combination of ICI and chemotherapy significantly prolonged overall survival when compared to chemotherapy alone (7, 8). However, patients with SCLC that has transformed from *EGFR*-mutated NSCLC constitute a relatively small population, and the efficacy of ICIs in such cases remains to be fully elucidated.

In this report, we describe a case of SCLC that transformed from *EGFR*-mutated NSCLC after EGFR-TKI treatment and achieved a long-term response with subsequent anti-PD-1 monotherapy.

## 2 Case presentation

**Figure 1:**
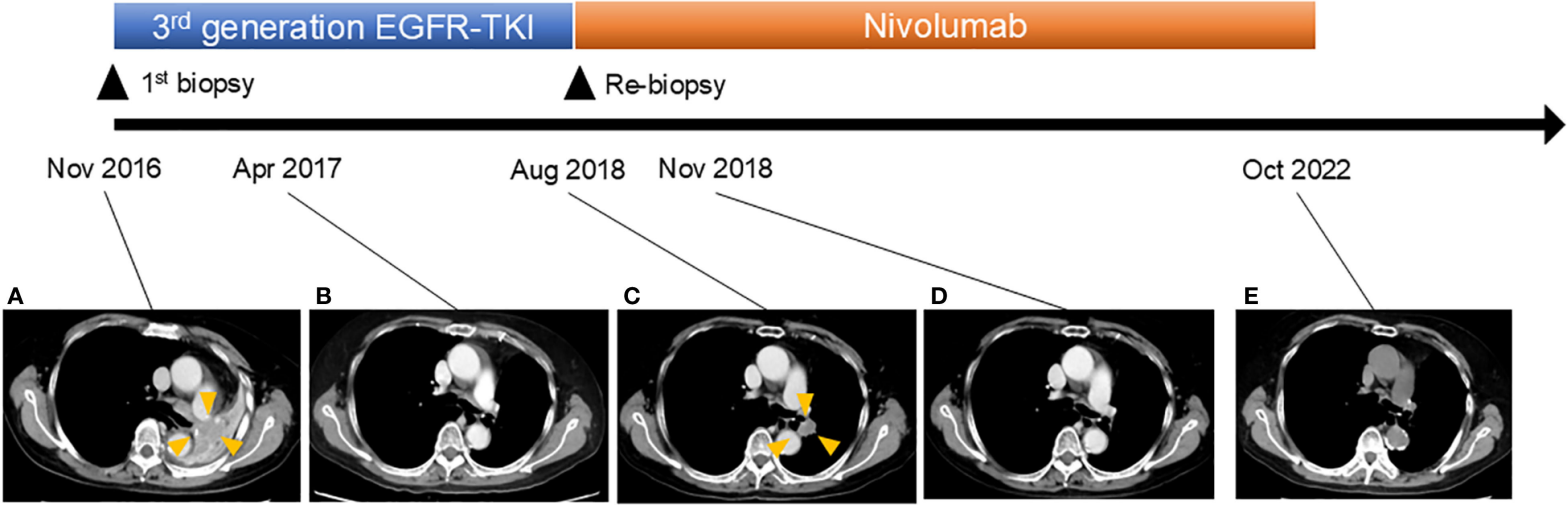
Clinical course of the patient. Computed tomography scan revealed the following: **(A)** Recurrence of lung cancer in the left pulmonary hilar region 9 years after surgery. **(B)** Disappearance of tumor after osimertinib treatment. **(C)** Regrowth of tumor. Osimertinib treatment was stopped and nivolumab treatment was initiated. **(D)** Disappearance of tumor after nivolumab treatment. **(E)** Continued tumor disappearance under nivolumab treatment.

**Figure 2:**
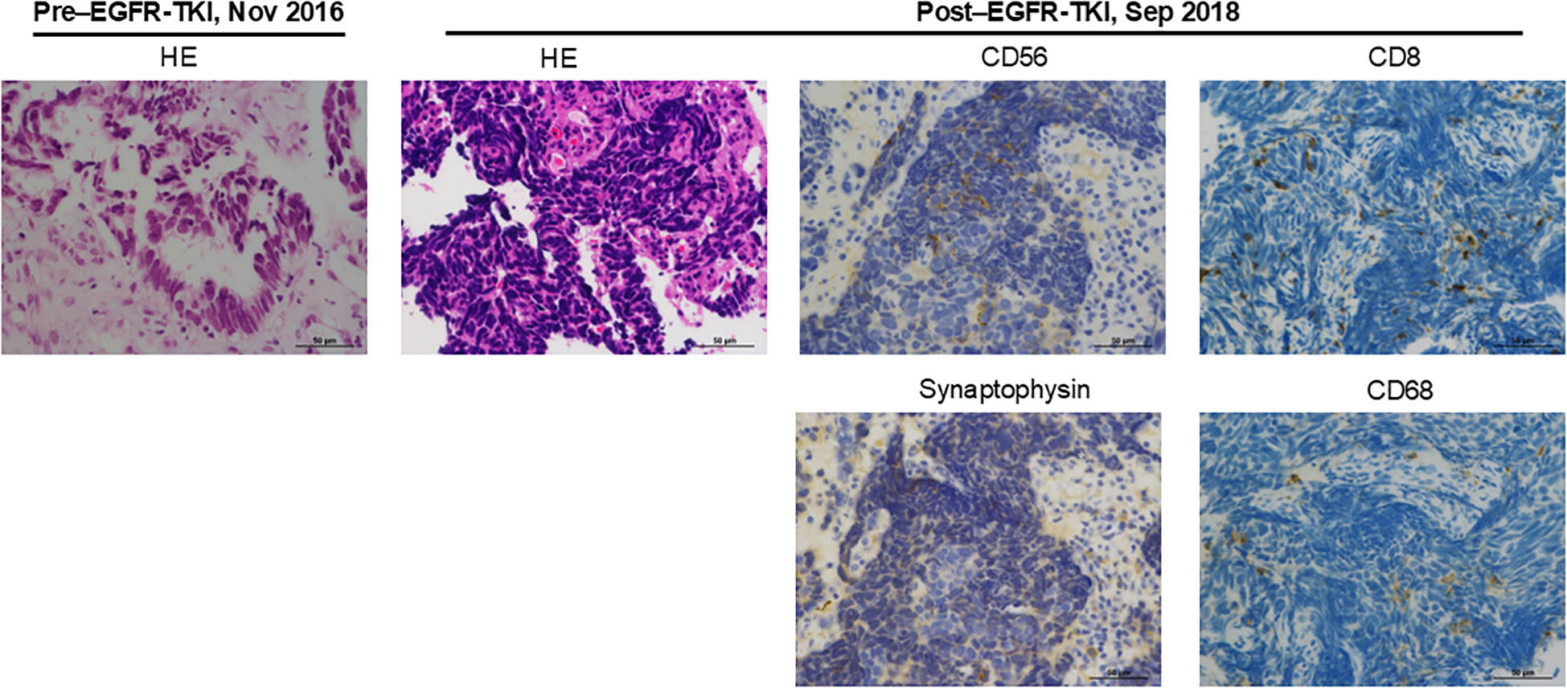
Pathologic examination of tumor obtained before EGFR-TKI treatment and after developing resistance to EGFR-TKI. The images show hematoxylin and eosin (HE) or immunohistochemical staining of CD56, synaptophysin, CD8, and CD68.

A 76-year-old woman presented to our clinic with the chief complaint of bloody sputum in November 2016. The patient had smoked 20 cigarettes per day for the past 25 years and had a medical history of left upper lobectomy for lung adenocarcinoma at another hospital in 2007. Chest and abdominal computed tomography (CT) images showed a metastatic lung tumor in the left pulmonary hilar region, multiple mediastinal lymph node metastases, and a rib metastasis (**Figure 1**). A bronchoscopic lung biopsy was performed for pathologic diagnosis. The final diagnosis was stage IVA (c-T3N2M1b) lung adenocarcinoma harboring an *EGFR* exon 19 deletion (**Figure 2**).

In December 2016, the patient was initiated on a third-generation EGFR-TKI, resulting in complete response to the treatment (**Figure 1**). However, in August 2018, EGFR-TKI was discontinued due to the recurrence of the tumor in the left lung (**Figure 1**). In order to obtain a pathological diagnosis of this exacerbated pulmonary tumor, a bronchoscopic re-biopsy was performed. The pathology results indicated a transformation to SCLC, with partly positive expression of CD56 and synaptophysin (**Figure 2**). Polymerase chain reaction testing showed that the *EGFR* exon 19 deletion remained positive, without secondary *EGFR* mutations detected. Additionally, programmed death-ligand 1 (PD-L1) tumor proportion score was negative (less than 1%).

In September 2018, the patient was enrolled in a clinical trial and was initiated on monotherapy with the anti-PD1 antibody nivolumab, 3 mg/kg, on day 1 every 2 weeks. Notably, 3 months later, in November 2018, CT imaging revealed left pulmonary tumor shrinkage. In May 2019, grade 3 adrenocorticotropic hormone deficiency was observed as an adverse event related to nivolumab treatment. However, nivolumab treatment was continued with corticosteroid replacement therapy. Subsequently, nivolumab treatment was continued until October 2022, when treatment was discontinued due to patient relocation. As of the end of 2023, there was no evidence of tumor progression.

## 3 Discussion

At the time this patient was treated with nivolumab, the efficacy of ICI in *EGFR*-mutated NSCLC was not clear, and nivolumab treatment was administered as a clinical trial. Currently, the efficacy of ICIs in *EGFR*-mutated NSCLC is generally considered limited. However, the efficacy of ICIs remains to be fully evaluated in the relatively small subpopulation of patients whose tumor has transformed to SCLC after developing resistance to EGFR-TKIs (9). The present case with a SCLC transformation showed a long-term response to anti-PD-1 despite the presence of a positive *EGFR* mutation. In other retrospective observation studies, there is a trend toward a better prognosis in patients with *EGFR*-mutated and transformed SCLC treated with ICI and chemotherapy compared to chemotherapy alone (10, 11). Specifically, the median overall survival was 10 months for patients treated with chemotherapy alone versus 13 months for patients treated with chemotherapy plus ICI in patients whose tumor transformed after developing resistance to EGFR-TKI therapy (Hazard ratio 0.75, 0.36−1.56) (10). Similarly, in patients with EGFR-mutated NSCLC that had transformed to SCLC, Zhang CY et al. reported that the median overall survival of patients who received immunotherapy was significantly longer than that of patients who did not receive immunotherapy (20.2 m versus 7.9 m, P < 0.01) (11). In contrast to these observations, other groups reported no long-term efficacy of ICI alone in their cohorts including cases of *EGFR*-mutated and transformed SCLC (12, 13). Specifically, Fujimoto D et al. reported only one case of response among 15 patients with *EGFR*-mutated NSCLC whose tumor has transformed to small cell lung cancer after developing resistance to EGFR-TKIs (12). Marcoux N et al. also reported no response among 17 patients with *EGFR*-mutated NSCLC who received ICI alone after SCLC-transformation (13). Collectively, the response rate to ICI alone may not necessarily be high in *EGFR*-mutated NSCLC, even with small cell transformation. However, it is noteworthy that ICI treatment may result in long-term survival in subpopulation with SCLC transformation, as evidenced in the present case report. Therefore, patient selection by biomarkers may be desirable for ICI treatment in patients with *EGFR*-mutated NSCLC with SCLC transformation.

**Figure 3:**
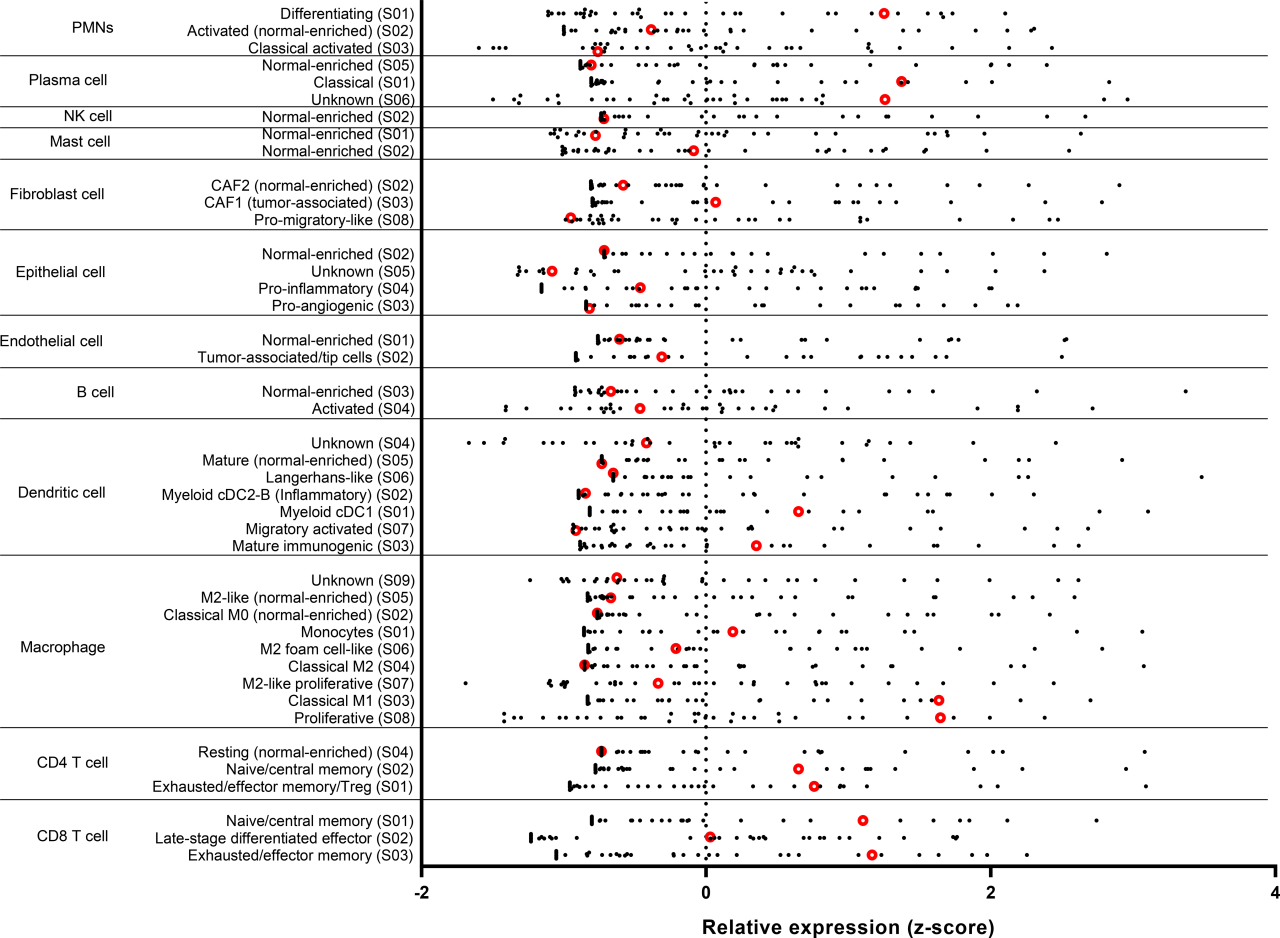
Tumor inflammatory cell profiles from 35 patients with EGFR-mutated NSCLC that has developed resistance to EGFR-TKIs. Inflammatory cell types were determined by CIBERSORTx analysis. Red dots indicate the present case, which has transformed to small cell lung cancer.

In general, tumor mutation burden (TMB) is relatively high in SCLC, partly due to heavy smoking. However, despite a smoking history, the TMB value is low in the present patient. While an association between TMB and the effect of ICI has been reported, the therapeutic effect of ICI in the present case is difficult to explain from TMB (14). The expression level of PD-L1 is also associated with the therapeutic effect of ICI in NSCLC and other types of cancer, but was negative in this case (15). Furthermore, an association between the infiltration of inflammatory cells into the tumor area and the therapeutic effect of ICI has been reported (16). We observed an infiltration of CD8- or CD68-positive immune cells in the current SCLC-transformed tumor (**Figure 2**). Additionally, for assessing the infiltration levels of multiple immune cells to estimate the tumor microenvironment, CIBERSORTx was performed using transcriptome data obtained from *EGFR*-mutated NSCLC tumor developing resistance to EGFR-TKIs (**Figure 3**, Supplementary Material and Methods) (17). Among 35 tumors obtained from patients with *EGFR*-mutated NSCLC treated with EGFR-TKIs, the present tumor showed relatively increased inflammatory cells, including exhausted CD4 and CD8 positive cells and M1-like macrophages, compared to other tumors (**Figure 3**). Consistent with the present case, Haratani K et al. reported that in patients with *EGFR*-mutated NSCLC, nivolumab responders had significantly higher CD8+ tumor-infiltrating lymphocyte (TIL) density than non-responders (18). Moreover, according to a retrospective analysis of extensive-stage SCLC, patients with high TIL demonstrated significantly superior progression-free survival compared to those with low TIL (19). Overall, ICI therapy may be beneficial in patients with *EGFR*-mutated NSCLC that has transformed into SCLC accompanied by CD8-positive TIL infiltration.

As a limitation, this is only a single case report of sustained response to ICI treatment, and further studies are needed to determine which patients would benefit most from ICI treatment after SCLC transformation based on clinical or genetic background. The combination of ICI and chemotherapy may also need continued investigation given its success in extensive-stage SCLC. Specifically, limited response rates to ICI monotherapy have been reported in extensive-stage SCLC, ranging from 2.3% for monotherapy with the anti-PD-L1 antibody atezolizumab to 9.5% for monotherapy with the anti-PD-L1 antibody durvalumab (20, 21). However, the combination of ICI and chemotherapy (i.e., platinum and etoposide) demonstrated a significant improvement in overall survival compared to chemotherapy alone in extensive-stage SCLC (7, 8).

## 4 Conclusion

ICI might be a treatment option in cases with *EGFR*-mutated and transformed SCLC. Further case series are required to evaluate the relationship between the tumor immune environment or the expression profile of immune checkpoint molecules and therapeutic response of ICI treatment.

## Data Availability

The datasets presented in this study can be found in online repositories. The names of the repository/repositories and accession number(s) can be found in the article/**Supplementary Material**.
