# Supplementary material for: Case Report: A small cell lung cancer transformed from an EGFR-mutated Adenocarcinoma demonstrated a long-term remission to anti PD-1 antibody
Source: Front Oncol. 2025 Sep 10;15:1651248. doi: 10.3389/fonc.2025.1651248 (PMC12457102; doi:10.3389/fonc.2025.1651248)
Supplement: Supplementary file 1 [file Table1.docx]

Supplementary Material

# Supplementary Data

**Supplementary Material and Methods**

**Transcriptional Profiling**

RNA was extracted from formalin-fixed paraffin-embedded (FFPE) tumors containing >50% cancer cells. The cDNA library for RNA-seq was prepared for the directional sequencing method with the ribosomal RNA depletion and the unique-dual indexes using the following kits and reagents: QIAseq FastSelect (Qiagen Inc., Venlo, Netherlands), NEBNext Ultra II Directional RNA Library Prep Kit for Illumina (New England Biolabs, Inc., Ipswich, MA, USA), and NEBNext Multiplex Oligos for Illumina (New England Biolabs, Inc.). We used an RNA input of 20 ng for library preparation; however, if the amount of RNA was insufficient, the maximum RNA input was used. The quantity of the prepared library was evaluated using the 4200 TapeStation. The prepared libraries were pooled in one tube and sequenced on one flow cell at 1.5 nM using a NovaSeq6000 (Illumina, Inc.), with 75 bp from both ends (2 × 75 bp). The sequencing reads were aligned with STAR software (2.5.3a) to the human genome reference (GRCh38). The transcripts per million and expected count for each gene were estimated using RNA-seq and expectation maximization (RSEM) software (1.3.0). Samples with non-cancer cells or a low sum of expected counts were excluded and normalized using the trimmed mean of the M value normalization method. For data analysis, samples with <50% within a species or total reads <50M were excluded.

**Tumor Microenvironment Analysis**

The infiltration levels of multiple immune cells were determined to estimate the tumor microenvironment (TME) in EGFR-mutated NSCLC using the Carcinoma EcoTyper web tool. The data were subsequently uploaded to the CIBERSORTx web portal, and the algorithm was run using the LM22 signature for 1000 permutations.
